# Bis(μ_2_-phenyl­tellurolato)bis­(phenyl­tellurolato)tetra-μ_3_-tellurido-hexa­kis(triphenyl­phosphine)hexa­palladium(II) benzene nona­solvate

**DOI:** 10.1107/S1600536810000206

**Published:** 2010-01-13

**Authors:** Maarit Risto, Raija Oilunkaniemi, Risto S. Laitinen, Markku Ahlgrén

**Affiliations:** a Department of Chemistry, PO Box 3000, FI-90014 University of Oulu, Finland; b Department of Chemistry, University of Joensuu, PO Box 111, FI-80101 Joensuu, Finland

## Abstract

The centrosymmetric title complex, [Pd_6_(C_6_H_5_Te)_4_Te_4_(C_18_H_15_P)_6_]·9C_6_H_6_, contains two Pd_3_Te_2_ cores that are joined into a cyclic hexa­nuclear complex by two bridging PhTe^−^ groups. Each Pd^II^ atom is coordinated by one triphenyl­phosphine ligand, one phenyl­tellurolate mol­ecule and two telluride ligands: two of the PhTe^−^ ligands act as terminal ligands and two as bridging ligands. The three distinct Pd^II^ atoms each show a slightly distorted PdPTe_3_ square-planar coordination. The asymmetric unit also contains four and a half benzene solvent mol­ecules. Two of the benzene mol­ecules are disordered: one mol­ecule is distributed over two positions with site-occupancy factors of 0.529 (7) and 0.471 (7), while the other occupies two orientations about a centre of symmetry.

## Related literature

For related complexes with a similar hexa­nuclear Pd_6_Te_6_ core, see: Oilunkaniemi *et al.* (2000 ▶, 2001 ▶); Brennan *et al.* (1990 ▶).
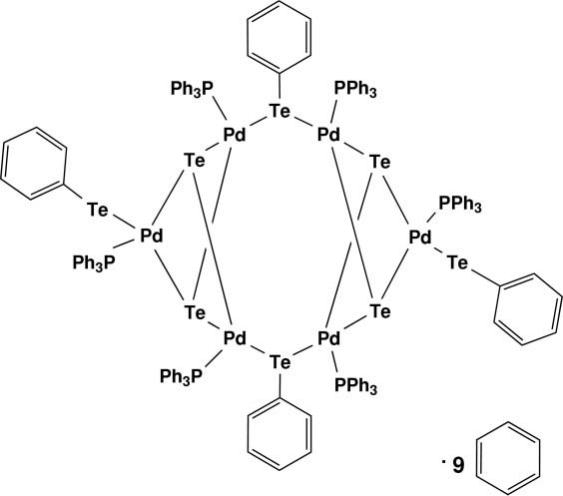


## Experimental

### 

#### Crystal data

[Pd_6_(C_6_H_5_Te)_4_Te_4_(C_18_H_15_P)_6_]·9C_6_H_6_
*M* *_r_* = 4244.19
Triclinic, 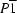

*a* = 16.8836 (3) Å
*b* = 17.2857 (3) Å
*c* = 18.1216 (3) Å
α = 81.908 (1)°
β = 62.385 (1)°
γ = 61.471 (1)°
*V* = 4095.06 (12) Å^3^
*Z* = 1
Mo *K*α radiation
μ = 2.15 mm^−1^
*T* = 120 K
0.25 × 0.15 × 0.15 mm

#### Data collection

Nonius KappaCCD diffractometer
Absorption correction: multi-scan (*SHELXTL*; Sheldrick, 2008 ▶) *T*_min_ = 0.615, *T*_max_ = 0.738
79007 measured reflections
16088 independent reflections
12698 reflections with *I* > 2σ(*I*)
*R*_int_ = 0.068

#### Refinement

*R*[*F* ^2^ > 2σ(*F* ^2^)] = 0.031
*wR*(*F* ^2^) = 0.062
*S* = 1.02
16088 reflections
938 parameters
H-atom parameters constrained
Δρ_max_ = 0.66 e Å^−3^
Δρ_min_ = −0.93 e Å^−3^

### 

Data collection: *COLLECT* (Nonius, 1998 ▶); cell refinement: *DENZO-SMN* (Otwinowski & Minor, 1997 ▶); data reduction: *DENZO-SMN*; program(s) used to solve structure: *SHELXS97* (Sheldrick, 2008 ▶); program(s) used to refine structure: *SHELXL97* (Sheldrick, 2008 ▶); molecular graphics: *DIAMOND* (Brandenburg & Berndt, 1999 ▶); software used to prepare material for publication: *WinGX* (Farrugia, 1999 ▶).

## Supplementary Material

Crystal structure: contains datablocks I, global. DOI: 10.1107/S1600536810000206/hb5293sup1.cif

Structure factors: contains datablocks I. DOI: 10.1107/S1600536810000206/hb5293Isup2.hkl

Additional supplementary materials:  crystallographic information; 3D view; checkCIF report

## Figures and Tables

**Table 1:** Selected bond lengths (Å)

Pd1—P1	2.2825 (9)
Pd1—Te4	2.5938 (4)
Pd1—Te3	2.6101 (3)
Pd1—Te1	2.6268 (4)
Pd2—P2	2.2937 (9)
Pd2—Te4	2.5928 (3)
Pd2—Te3	2.6101 (3)
Pd2—Te2	2.6389 (4)
Pd3—P3	2.3001 (9)
Pd3—Te4	2.5928 (3)
Pd3—Te1^i^	2.6124 (3)
Pd3—Te3	2.6125 (3)

Symmetry code: (i) 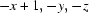.

**Fig. 1.**
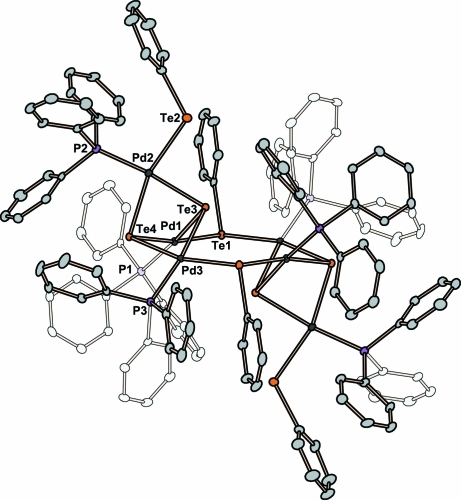
The molecular structure of (I) showing 50% displacement ellipsoids. All hydrogen atoms and the solvent molecules are omitted for clarity.

**Fig. 2.**
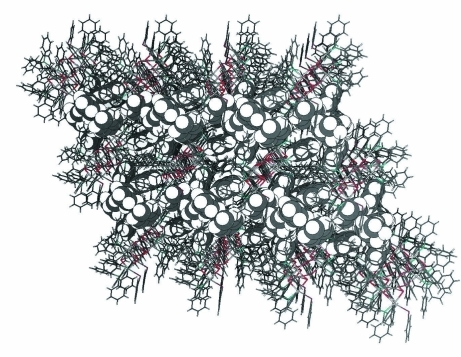
The packing of the molecules showing the alternating layers of the complex and that of the solvent molecules. Some of the benzene molecules are disordered.

## supplementary crystallographic information

### Comment

The structure of (I) (Fig. 1), is composed of two Pd_3_Te_2_ fragments joined together into a cyclic hexanuclear complex by two bridging PhTe^-^ ligands. A similar hexanuclear structure has been reported for [Pd_6_Te_4_{Te(C_4_H_3_S)}_4_(PPh_3_)_6_] (Oilunkaniemi *et al.*, 2000). The compound (I) also shows the same Pd_3_Te_2_ framework as [Pd_6_Cl_2_Te_4_(TePh)_2_(PPh_3_)_6_] (Oilunkaniemi *et al.*, 2001), [Pd_6_Cl_2_Te_4_{Te(C_4_H_3_S)}_2_(PPh_3_)_6_] (Oilunkaniemi *et al.*, 2000), and [Pd_6_Te_6_(PEt_3_)_8_] (Brennan *et al.*, 1990).

The packing of the molecules is shown in Fig. 2. The lattice shows layers of solvent molecules (35% of the cell volume is occupied by the solvent) some of which are disordered that separate layers containing the complex molecules.

### Experimental

Ph_2_Te_2_ (0.185 g, 0.452 mmol) in 5 ml of methanol was treated with NaBH_4_ until the solution became colourless. The solution was added to a suspension of [PdCl_2_(PPh_3_)_2_] (0.298 g, 0.425 mmol) in 10 ml benzene. The reaction mixture was stirred overnight at room temperature. Volatile materials were removed under dynamic vacuum and the residue was dissolved in benzene (10 ml). After filtration the solution was concentrated by partial evaporation of the solvent. A small amount of red plates of (I) were formed upon standing of the solution at room temperature.

### Refinement

H atoms were positioned geometrically and treated as riding, with C—H = 0.95 Å and with *U*_iso_(H) = 1.2*U*_eq_(C). The site occupancy factors of one of the two disordered benzene molecules were refined by constraining the anisotropic displacement factors in the disordered pairs of carbon atoms as equal. The other disordered molecule occupies two orientations about the centre of symmetry and therefore its site occupancy factor was fixed at 0.5.

### Crystal data

**Table:**

[Pd_6_(C_6_H_5_Te)_4_Te_4_(C_18_H_15_P)_6_]·9C_6_H_6_	*Z* = 1
*M**_r_* = 4244.19	*F*(000) = 2062
Triclinic, *P*1	*D*_x_ = 1.721 Mg m^−^^3^
Hall symbol: -P 1	Mo *K*α radiation, λ = 0.71073 Å
*a* = 16.8836 (3) Å	Cell parameters from 12698 reflections
*b* = 17.2857 (3) Å	θ = 2.8–26.0°
*c* = 18.1216 (3) Å	µ = 2.15 mm^−^^1^
α = 81.908 (1)°	*T* = 120 K
β = 62.385 (1)°	Plate, red
γ = 61.471 (1)°	0.25 × 0.15 × 0.15 mm
*V* = 4095.06 (12) Å^3^	

### Data collection

**Table:**

Nonius KappaCCD diffractometer	16088 independent reflections
Radiation source: fine-focus sealed tube	12698 reflections with *I* > 2σ(*I*)
graphite	*R*_int_ = 0.068
φ scans, and ω scans with κ offsets	θ_max_ = 26.0°, θ_min_ = 2.8°
Absorption correction: ψ scan (*SHELXTL*; Sheldrick, 2008)	*h* = −20→20
*T*_min_ = 0.615, *T*_max_ = 0.738	*k* = −21→21
79007 measured reflections	*l* = −22→22

### Refinement

**Table:**

Refinement on *F*^2^	Primary atom site location: structure-invariant direct methods
Least-squares matrix: full	Secondary atom site location: difference Fourier map
*R*[*F*^2^ > 2σ(*F*^2^)] = 0.031	Hydrogen site location: inferred from neighbouring sites
*wR*(*F*^2^) = 0.062	H-atom parameters constrained
*S* = 1.01	*w* = 1/[σ^2^(*F*_o_^2^) + (0.0232*P*)^2^ + 2.3871*P*] where *P* = (*F*_o_^2^ + 2*F*_c_^2^)/3
16088 reflections	(Δ/σ)_max_ = 0.001
938 parameters	Δρ_max_ = 0.66 e Å^−^^3^
0 restraints	Δρ_min_ = −0.93 e Å^−^^3^

### Special details

**Table:**

Geometry. All e.s.d.'s (except the e.s.d. in the dihedral angle between two l.s. planes) are estimated using the full covariance matrix. The cell e.s.d.'s are taken into account individually in the estimation of e.s.d.'s in distances, angles and torsion angles; correlations between e.s.d.'s in cell parameters are only used when they are defined by crystal symmetry. An approximate (isotropic) treatment of cell e.s.d.'s is used for estimating e.s.d.'s involving l.s. planes.
Refinement. Refinement of *F*^2^ against ALL reflections. The weighted *R*-factor *wR* and goodness of fit *S* are based on *F*^2^, conventional *R*-factors *R* are based on *F*, with *F* set to zero for negative *F*^2^. The threshold expression of *F*^2^ > σ(*F*^2^) is used only for calculating *R*-factors(gt) *etc*. and is not relevant to the choice of reflections for refinement. *R*-factors based on *F*^2^ are statistically about twice as large as those based on *F*, and *R*- factors based on ALL data will be even larger.

### Fractional atomic coordinates and isotropic or equivalent isotropic displacement parameters (Å^2^)

**Table:**

	*x*	*y*	*z*	*U*_iso_*/*U*_eq_	Occ. (<1)
Pd1	0.344230 (19)	0.152782 (17)	0.025197 (17)	0.01242 (6)	
Pd2	0.259693 (19)	0.173945 (17)	0.238351 (18)	0.01449 (7)	
Pd3	0.333793 (18)	−0.026436 (16)	0.110705 (17)	0.01212 (6)	
Te1	0.500965 (16)	0.175025 (14)	−0.081148 (15)	0.01468 (6)	
Te2	0.377353 (19)	0.160500 (17)	0.302342 (18)	0.02665 (7)	
Te3	0.425242 (16)	0.055640 (14)	0.119977 (15)	0.01252 (6)	
Te4	0.194736 (16)	0.137658 (14)	0.150057 (15)	0.01342 (6)	
P1	0.25377 (7)	0.23743 (6)	−0.04461 (6)	0.0158 (2)	
P2	0.10855 (7)	0.29098 (6)	0.32256 (6)	0.0172 (2)	
P3	0.23063 (7)	−0.07616 (6)	0.10816 (6)	0.0153 (2)	
C11	0.4876 (2)	0.2676 (2)	−0.0037 (2)	0.0196 (9)	
C12	0.4695 (3)	0.3513 (2)	−0.0299 (3)	0.0258 (10)	
H12	0.4624	0.3657	−0.0798	0.031*	
C13	0.4617 (3)	0.4138 (3)	0.0169 (3)	0.0347 (11)	
H13	0.4475	0.4715	−0.0002	0.042*	
C14	0.4744 (3)	0.3928 (3)	0.0880 (3)	0.0365 (12)	
H14	0.4720	0.4349	0.1186	0.044*	
C15	0.4909 (3)	0.3097 (3)	0.1147 (3)	0.0341 (11)	
H15	0.4985	0.2953	0.1644	0.041*	
C16	0.4963 (3)	0.2478 (3)	0.0693 (3)	0.0252 (9)	
H16	0.5061	0.1914	0.0886	0.030*	
C21	0.3106 (3)	0.2691 (3)	0.3930 (3)	0.0272 (10)	
C22	0.3263 (3)	0.3421 (3)	0.3683 (3)	0.0364 (11)	
H22	0.3628	0.3459	0.3105	0.044*	
C23	0.2895 (4)	0.4093 (3)	0.4264 (3)	0.0478 (13)	
H23	0.3003	0.4593	0.4081	0.057*	
C24	0.2374 (4)	0.4053 (3)	0.5106 (3)	0.0466 (13)	
H24	0.2133	0.4513	0.5505	0.056*	
C25	0.2211 (4)	0.3336 (3)	0.5356 (3)	0.0481 (13)	
H25	0.1858	0.3297	0.5936	0.058*	
C26	0.2565 (3)	0.2633 (3)	0.4753 (2)	0.0255 (9)	
H26	0.2424	0.2149	0.4926	0.031*	
C111	0.3244 (3)	0.2180 (2)	−0.1592 (2)	0.0189 (8)	
C112	0.3795 (3)	0.1300 (3)	−0.1946 (3)	0.0234 (9)	
H112	0.3817	0.0837	−0.1592	0.028*	
C113	0.4307 (3)	0.1097 (3)	−0.2804 (3)	0.0307 (10)	
H113	0.4663	0.0499	−0.3036	0.037*	
C114	0.4302 (3)	0.1762 (3)	−0.3325 (3)	0.0356 (11)	
H114	0.4640	0.1624	−0.3915	0.043*	
C115	0.3806 (3)	0.2624 (3)	−0.2985 (3)	0.0358 (11)	
H115	0.3824	0.3080	−0.3344	0.043*	
C116	0.3282 (3)	0.2835 (3)	−0.2126 (3)	0.0252 (9)	
H116	0.2945	0.3433	−0.1900	0.030*	
C121	0.1400 (3)	0.2302 (2)	−0.0226 (2)	0.0159 (8)	
C122	0.1400 (3)	0.1772 (2)	−0.0733 (2)	0.0211 (9)	
H122	0.2002	0.1443	−0.1225	0.025*	
C123	0.0530 (3)	0.1717 (3)	−0.0527 (3)	0.0246 (9)	
H123	0.0542	0.1347	−0.0878	0.029*	
C124	−0.0347 (3)	0.2192 (2)	0.0177 (3)	0.0220 (9)	
H124	−0.0945	0.2164	0.0307	0.026*	
C125	−0.0357 (3)	0.2711 (2)	0.0698 (3)	0.0214 (9)	
H125	−0.0964	0.3038	0.1189	0.026*	
C126	0.0512 (3)	0.2758 (2)	0.0509 (2)	0.0168 (8)	
H126	0.0506	0.3101	0.0880	0.020*	
C131	0.2042 (2)	0.3545 (2)	−0.0188 (2)	0.0166 (8)	
C132	0.1424 (3)	0.4190 (2)	−0.0508 (3)	0.0248 (9)	
H132	0.1263	0.4020	−0.0876	0.030*	
C133	0.1040 (3)	0.5085 (3)	−0.0292 (3)	0.0312 (10)	
H133	0.0630	0.5523	−0.0521	0.037*	
C134	0.1256 (3)	0.5335 (3)	0.0252 (3)	0.0300 (10)	
H134	0.0992	0.5946	0.0398	0.036*	
C135	0.1848 (3)	0.4710 (3)	0.0584 (3)	0.0300 (10)	
H135	0.1988	0.4885	0.0964	0.036*	
C136	0.2245 (3)	0.3816 (2)	0.0362 (2)	0.0230 (9)	
H136	0.2661	0.3385	0.0590	0.028*	
C211	0.0053 (3)	0.3386 (2)	0.2928 (2)	0.0212 (9)	
C212	−0.0509 (3)	0.2942 (3)	0.3064 (2)	0.0262 (9)	
H212	−0.0323	0.2378	0.3269	0.031*	
C213	−0.1335 (3)	0.3326 (3)	0.2900 (3)	0.0360 (11)	
H213	−0.1716	0.3025	0.3002	0.043*	
C214	−0.1610 (3)	0.4139 (3)	0.2591 (3)	0.0386 (12)	
H214	−0.2186	0.4403	0.2491	0.046*	
C215	−0.1051 (3)	0.4562 (3)	0.2431 (3)	0.0399 (12)	
H215	−0.1228	0.5113	0.2201	0.048*	
C216	−0.0222 (3)	0.4197 (3)	0.2599 (3)	0.0292 (10)	
H216	0.0156	0.4503	0.2489	0.035*	
C221	0.0433 (3)	0.2723 (2)	0.4310 (2)	0.0194 (8)	
C222	−0.0607 (3)	0.3235 (2)	0.4812 (2)	0.0233 (9)	
H222	−0.1001	0.3716	0.4599	0.028*	
C223	−0.1062 (3)	0.3038 (3)	0.5624 (3)	0.0285 (10)	
H223	−0.1769	0.3388	0.5963	0.034*	
C224	−0.0510 (3)	0.2349 (3)	0.5945 (3)	0.0317 (10)	
H224	−0.0834	0.2223	0.6501	0.038*	
C225	0.0520 (3)	0.1836 (3)	0.5458 (3)	0.0337 (10)	
H225	0.0906	0.1364	0.5684	0.040*	
C226	0.0992 (3)	0.2008 (3)	0.4637 (3)	0.0262 (9)	
H226	0.1694	0.1640	0.4298	0.031*	
C231	0.1226 (3)	0.3888 (2)	0.3248 (2)	0.0186 (8)	
C232	0.2020 (3)	0.3955 (3)	0.2568 (2)	0.0237 (9)	
H232	0.2519	0.3467	0.2152	0.028*	
C233	0.2095 (3)	0.4732 (3)	0.2488 (3)	0.0265 (9)	
H233	0.2638	0.4779	0.2015	0.032*	
C234	0.1377 (3)	0.5435 (3)	0.3099 (3)	0.0266 (9)	
H234	0.1420	0.5970	0.3046	0.032*	
C235	0.0598 (3)	0.5364 (3)	0.3786 (3)	0.0269 (9)	
H235	0.0113	0.5848	0.4209	0.032*	
C236	0.0513 (3)	0.4607 (2)	0.3867 (2)	0.0236 (9)	
H236	−0.0030	0.4567	0.4344	0.028*	
C311	0.0970 (2)	−0.0132 (2)	0.1846 (2)	0.0163 (8)	
C312	0.0308 (3)	0.0606 (2)	0.1626 (3)	0.0204 (8)	
H312	0.0534	0.0750	0.1065	0.025*	
C313	−0.0675 (3)	0.1131 (3)	0.2218 (3)	0.0259 (9)	
H313	−0.1124	0.1627	0.2060	0.031*	
C314	−0.1002 (3)	0.0936 (3)	0.3032 (3)	0.0355 (11)	
H314	−0.1677	0.1299	0.3437	0.043*	
C315	−0.0355 (3)	0.0216 (3)	0.3268 (3)	0.0368 (11)	
H315	−0.0585	0.0086	0.3834	0.044*	
C316	0.0626 (3)	−0.0314 (3)	0.2680 (3)	0.0263 (9)	
H316	0.1070	−0.0806	0.2845	0.032*	
C321	0.2257 (3)	−0.0698 (2)	0.0087 (2)	0.0194 (8)	
C322	0.1511 (3)	−0.0774 (2)	0.0009 (3)	0.0220 (9)	
H322	0.0998	−0.0854	0.0486	0.026*	
C323	0.1520 (3)	−0.0733 (3)	−0.0765 (3)	0.0272 (10)	
H323	0.0998	−0.0764	−0.0812	0.033*	
C324	0.2282 (3)	−0.0647 (3)	−0.1466 (3)	0.0292 (10)	
H324	0.2299	−0.0641	−0.1998	0.035*	
C325	0.3021 (3)	−0.0570 (3)	−0.1392 (3)	0.0278 (10)	
H325	0.3544	−0.0509	−0.1874	0.033*	
C326	0.3002 (3)	−0.0581 (2)	−0.0620 (2)	0.0215 (9)	
H326	0.3499	−0.0508	−0.0571	0.026*	
C331	0.2620 (3)	−0.1905 (2)	0.1336 (3)	0.0197 (9)	
C332	0.2848 (3)	−0.2164 (2)	0.1997 (2)	0.0227 (9)	
H332	0.2843	−0.1750	0.2296	0.027*	
C333	0.3085 (3)	−0.3014 (3)	0.2238 (3)	0.0287 (10)	
H333	0.3239	−0.3181	0.2697	0.034*	
C334	0.3095 (3)	−0.3612 (3)	0.1803 (3)	0.0343 (11)	
H334	0.3243	−0.4191	0.1970	0.041*	
C335	0.2891 (3)	−0.3377 (3)	0.1128 (3)	0.0358 (12)	
H335	0.2917	−0.3800	0.0824	0.043*	
C336	0.2648 (3)	−0.2523 (2)	0.0887 (3)	0.0259 (10)	
H336	0.2502	−0.2361	0.0424	0.031*	
C31	0.1641 (2)	0.8741 (2)	0.44996 (18)	0.0461 (13)	
H31	0.1082	0.8662	0.4908	0.055*	
C32	0.14849 (19)	0.95676 (19)	0.4218 (2)	0.0474 (13)	
H32	0.0820	1.0053	0.4433	0.057*	
C33	0.2302 (3)	0.9684 (2)	0.3621 (2)	0.0468 (13)	
H33	0.2195	1.0249	0.3428	0.056*	
C34	0.3274 (2)	0.8973 (3)	0.33059 (18)	0.0552 (15)	
H34	0.3832	0.9053	0.2898	0.066*	
C35	0.34296 (19)	0.8146 (2)	0.3588 (2)	0.0614 (17)	
H35	0.4094	0.7661	0.3372	0.074*	
C36	0.2613 (3)	0.80304 (18)	0.4185 (2)	0.0559 (15)	
H36	0.2719	0.7465	0.4377	0.067*	
C41	0.8429 (3)	0.7635 (4)	0.2667 (3)	0.085 (3)	
H41	0.7896	0.7500	0.2805	0.102*	
C42	0.9431 (3)	0.6978 (3)	0.2279 (3)	0.0702 (18)	
H42	0.9583	0.6393	0.2153	0.084*	
C43	1.0211 (2)	0.7176 (2)	0.2077 (2)	0.0509 (14)	
H43	1.0895	0.6727	0.1812	0.061*	
C44	0.9989 (2)	0.8031 (2)	0.2262 (2)	0.0459 (13)	
H44	1.0522	0.8166	0.2123	0.055*	
C45	0.8987 (3)	0.8688 (2)	0.2649 (2)	0.0641 (17)	
H45	0.8836	0.9273	0.2776	0.077*	
C46	0.82076 (18)	0.8490 (3)	0.2852 (2)	0.086 (3)	
H46	0.7523	0.8940	0.3117	0.104*	
C61	0.6481 (5)	0.3470 (3)	0.1813 (5)	0.111 (3)	
H61	0.6751	0.2874	0.1615	0.133*	
C62	0.6851 (3)	0.4013 (4)	0.1289 (3)	0.081 (2)	
H62	0.7374	0.3788	0.0734	0.097*	
C63	0.6455 (3)	0.4886 (4)	0.1578 (3)	0.078 (2)	
H63	0.6708	0.5257	0.1221	0.094*	
C64	0.5690 (4)	0.5215 (4)	0.2391 (4)	0.109 (3)	
H64	0.5419	0.5812	0.2589	0.131*	
C65	0.5319 (4)	0.4672 (6)	0.2914 (2)	0.145 (5)	
H65	0.4796	0.4898	0.3470	0.174*	
C66	0.5715 (5)	0.3800 (5)	0.2625 (4)	0.153 (5)	
H66	0.5462	0.3428	0.2983	0.184*	
C51A	0.5375 (5)	0.0513 (5)	0.4413 (5)	0.068 (4)	0.529 (7)
H51A	0.4891	0.0438	0.4342	0.082*	0.529 (7)
C52A	0.5848 (5)	−0.0054 (4)	0.4873 (4)	0.056 (3)	0.529 (7)
H52A	0.5689	−0.0516	0.5116	0.067*	0.529 (7)
C53A	0.6555 (5)	0.0056 (4)	0.4977 (4)	0.042 (3)	0.529 (7)
H53A	0.6879	−0.0331	0.5291	0.051*	0.529 (7)
C54A	0.6789 (5)	0.0733 (5)	0.4621 (5)	0.045 (4)	0.529 (7)
H54A	0.7272	0.0808	0.4692	0.055*	0.529 (7)
C55A	0.6315 (6)	0.1300 (5)	0.4161 (5)	0.065 (4)	0.529 (7)
H55A	0.6475	0.1763	0.3918	0.078*	0.529 (7)
C56A	0.5608 (5)	0.1190 (5)	0.4057 (5)	0.063 (4)	0.529 (7)
H56A	0.5285	0.1577	0.3743	0.076*	0.529 (7)
C51B	0.6639 (6)	0.0341 (5)	0.4321 (5)	0.055 (3)	0.471 (7)
H51B	0.6940	−0.0273	0.4382	0.066*	0.471 (7)
C52B	0.6661 (6)	0.0928 (6)	0.4753 (5)	0.056 (6)	0.471 (7)
H52B	0.6978	0.0715	0.5109	0.067*	0.471 (7)
C53B	0.6219 (7)	0.1828 (6)	0.4664 (5)	0.054 (3)	0.471 (7)
H53B	0.6235	0.2229	0.4960	0.065*	0.471 (7)
C54B	0.5756 (6)	0.2140 (5)	0.4144 (5)	0.065 (4)	0.471 (7)
H54B	0.5454	0.2755	0.4083	0.078*	0.471 (7)
C55B	0.5734 (7)	0.1553 (6)	0.3711 (5)	0.070 (5)	0.471 (7)
H55B	0.5417	0.1766	0.3356	0.084*	0.471 (7)
C56B	0.6175 (7)	0.0653 (5)	0.3800 (6)	0.064 (4)	0.471 (7)
H56B	0.6160	0.0252	0.3505	0.077*	0.471 (7)
C71	0.5776 (15)	0.4406 (12)	0.4774 (14)	0.101 (9)	0.50
H71	0.6368	0.3849	0.4627	0.122*	0.50
C72	0.483 (2)	0.4444 (14)	0.5128 (14)	0.117 (8)	0.50
H72	0.4774	0.3913	0.5223	0.140*	0.50
C73	0.3963 (14)	0.526 (2)	0.5343 (14)	0.170 (15)	0.50
H73	0.3316	0.5284	0.5585	0.204*	0.50
C74	0.404 (2)	0.6035 (13)	0.5204 (17)	0.162 (16)	0.50
H74	0.3451	0.6591	0.5351	0.195*	0.50
C75	0.499 (3)	0.5997 (13)	0.4850 (18)	0.190 (19)	0.50
H75	0.5045	0.6528	0.4755	0.228*	0.50
C76	0.5856 (19)	0.5183 (19)	0.4635 (15)	0.134 (13)	0.50
H76	0.6503	0.5157	0.4393	0.160*	0.50

### Atomic displacement parameters (Å^2^)

**Table:**

	*U*^11^	*U*^22^	*U*^33^	*U*^12^	*U*^13^	*U*^23^
Pd1	0.01143 (13)	0.01042 (13)	0.01477 (16)	−0.00477 (11)	−0.00602 (11)	0.00185 (11)
Pd2	0.01403 (14)	0.01323 (14)	0.01385 (16)	−0.00471 (11)	−0.00557 (12)	−0.00152 (11)
Pd3	0.01160 (13)	0.00993 (13)	0.01485 (15)	−0.00530 (11)	−0.00557 (11)	0.00048 (11)
Te1	0.01305 (12)	0.01131 (12)	0.01799 (14)	−0.00570 (10)	−0.00615 (10)	0.00263 (10)
Te2	0.02369 (14)	0.02841 (15)	0.02676 (16)	−0.00652 (12)	−0.01439 (12)	−0.00536 (12)
Te3	0.01210 (11)	0.01060 (11)	0.01492 (13)	−0.00493 (9)	−0.00642 (10)	0.00059 (9)
Te4	0.01161 (11)	0.01150 (11)	0.01544 (13)	−0.00481 (9)	−0.00512 (10)	−0.00016 (10)
P1	0.0149 (5)	0.0137 (5)	0.0178 (5)	−0.0052 (4)	−0.0086 (4)	0.0026 (4)
P2	0.0172 (5)	0.0153 (5)	0.0152 (5)	−0.0060 (4)	−0.0056 (4)	−0.0013 (4)
P3	0.0151 (5)	0.0139 (5)	0.0185 (5)	−0.0090 (4)	−0.0066 (4)	0.0017 (4)
C11	0.0094 (18)	0.0136 (18)	0.027 (2)	−0.0042 (15)	−0.0011 (16)	−0.0049 (17)
C12	0.017 (2)	0.016 (2)	0.039 (3)	−0.0090 (17)	−0.0075 (18)	0.0020 (18)
C13	0.022 (2)	0.012 (2)	0.060 (4)	−0.0071 (17)	−0.010 (2)	−0.004 (2)
C14	0.028 (2)	0.028 (2)	0.045 (3)	−0.019 (2)	0.002 (2)	−0.020 (2)
C15	0.033 (2)	0.045 (3)	0.025 (3)	−0.026 (2)	−0.0024 (19)	−0.009 (2)
C16	0.022 (2)	0.024 (2)	0.026 (2)	−0.0139 (18)	−0.0048 (18)	−0.0016 (18)
C21	0.026 (2)	0.032 (2)	0.026 (3)	−0.0115 (19)	−0.0142 (19)	−0.0031 (19)
C22	0.039 (3)	0.037 (3)	0.031 (3)	−0.017 (2)	−0.014 (2)	−0.002 (2)
C23	0.058 (3)	0.042 (3)	0.043 (3)	−0.020 (3)	−0.025 (3)	−0.005 (3)
C24	0.053 (3)	0.036 (3)	0.040 (3)	−0.010 (2)	−0.019 (3)	−0.015 (2)
C25	0.054 (3)	0.059 (3)	0.021 (3)	−0.022 (3)	−0.010 (2)	−0.006 (2)
C26	0.021 (2)	0.026 (2)	0.019 (2)	−0.0100 (18)	−0.0023 (17)	0.0037 (18)
C111	0.0129 (18)	0.025 (2)	0.019 (2)	−0.0080 (16)	−0.0087 (16)	0.0024 (17)
C112	0.021 (2)	0.026 (2)	0.024 (2)	−0.0114 (18)	−0.0108 (18)	0.0043 (18)
C113	0.017 (2)	0.036 (2)	0.030 (3)	−0.0062 (19)	−0.0062 (19)	−0.010 (2)
C114	0.026 (2)	0.059 (3)	0.017 (2)	−0.021 (2)	−0.0040 (19)	0.000 (2)
C115	0.036 (2)	0.052 (3)	0.024 (3)	−0.027 (2)	−0.013 (2)	0.015 (2)
C116	0.026 (2)	0.028 (2)	0.025 (2)	−0.0160 (18)	−0.0118 (19)	0.0091 (19)
C121	0.0157 (18)	0.0114 (18)	0.019 (2)	−0.0055 (15)	−0.0092 (16)	0.0063 (16)
C122	0.020 (2)	0.023 (2)	0.017 (2)	−0.0084 (17)	−0.0069 (17)	−0.0003 (17)
C123	0.026 (2)	0.025 (2)	0.029 (2)	−0.0134 (18)	−0.0161 (19)	0.0038 (19)
C124	0.020 (2)	0.023 (2)	0.029 (2)	−0.0117 (17)	−0.0151 (18)	0.0092 (18)
C125	0.0165 (19)	0.0179 (19)	0.023 (2)	−0.0050 (16)	−0.0082 (17)	0.0041 (17)
C126	0.0219 (19)	0.0118 (18)	0.018 (2)	−0.0071 (16)	−0.0117 (17)	0.0057 (16)
C131	0.0130 (18)	0.0133 (18)	0.020 (2)	−0.0050 (15)	−0.0056 (16)	0.0013 (16)
C132	0.024 (2)	0.022 (2)	0.028 (2)	−0.0086 (18)	−0.0132 (18)	0.0022 (18)
C133	0.027 (2)	0.018 (2)	0.044 (3)	−0.0056 (18)	−0.018 (2)	0.009 (2)
C134	0.030 (2)	0.014 (2)	0.038 (3)	−0.0061 (18)	−0.013 (2)	0.0009 (19)
C135	0.039 (2)	0.024 (2)	0.028 (3)	−0.014 (2)	−0.016 (2)	0.0013 (19)
C136	0.023 (2)	0.018 (2)	0.025 (2)	−0.0072 (17)	−0.0131 (18)	0.0071 (17)
C211	0.0160 (19)	0.019 (2)	0.015 (2)	−0.0004 (16)	−0.0022 (16)	−0.0067 (16)
C212	0.024 (2)	0.027 (2)	0.022 (2)	−0.0069 (18)	−0.0083 (18)	−0.0075 (18)
C213	0.023 (2)	0.046 (3)	0.036 (3)	−0.010 (2)	−0.012 (2)	−0.016 (2)
C214	0.028 (2)	0.036 (3)	0.038 (3)	0.005 (2)	−0.020 (2)	−0.014 (2)
C215	0.049 (3)	0.027 (2)	0.041 (3)	−0.004 (2)	−0.032 (2)	0.001 (2)
C216	0.031 (2)	0.028 (2)	0.023 (2)	−0.0064 (19)	−0.0138 (19)	−0.0029 (19)
C221	0.023 (2)	0.0186 (19)	0.017 (2)	−0.0109 (17)	−0.0073 (17)	−0.0014 (16)
C222	0.028 (2)	0.020 (2)	0.020 (2)	−0.0134 (18)	−0.0055 (18)	−0.0036 (17)
C223	0.027 (2)	0.030 (2)	0.023 (2)	−0.0169 (19)	−0.0017 (19)	−0.0054 (19)
C224	0.046 (3)	0.038 (3)	0.019 (2)	−0.030 (2)	−0.011 (2)	0.006 (2)
C225	0.039 (3)	0.039 (3)	0.024 (3)	−0.023 (2)	−0.012 (2)	0.011 (2)
C226	0.028 (2)	0.028 (2)	0.024 (2)	−0.0142 (19)	−0.0117 (19)	0.0013 (19)
C231	0.023 (2)	0.0162 (19)	0.016 (2)	−0.0100 (16)	−0.0077 (17)	0.0015 (16)
C232	0.023 (2)	0.023 (2)	0.019 (2)	−0.0091 (17)	−0.0056 (17)	−0.0026 (17)
C233	0.029 (2)	0.030 (2)	0.023 (2)	−0.0205 (19)	−0.0078 (19)	0.0042 (19)
C234	0.043 (3)	0.022 (2)	0.023 (2)	−0.022 (2)	−0.015 (2)	0.0046 (18)
C235	0.031 (2)	0.019 (2)	0.023 (2)	−0.0099 (18)	−0.0066 (19)	−0.0046 (18)
C236	0.022 (2)	0.022 (2)	0.017 (2)	−0.0083 (17)	−0.0022 (17)	−0.0028 (17)
C311	0.0132 (18)	0.0157 (18)	0.023 (2)	−0.0108 (15)	−0.0055 (16)	0.0006 (16)
C312	0.021 (2)	0.024 (2)	0.021 (2)	−0.0138 (17)	−0.0095 (17)	0.0030 (17)
C313	0.017 (2)	0.023 (2)	0.033 (3)	−0.0064 (17)	−0.0102 (19)	−0.0013 (19)
C314	0.019 (2)	0.024 (2)	0.034 (3)	−0.0049 (19)	0.0053 (19)	−0.003 (2)
C315	0.036 (3)	0.028 (2)	0.021 (3)	−0.010 (2)	0.001 (2)	0.004 (2)
C316	0.027 (2)	0.021 (2)	0.023 (2)	−0.0116 (18)	−0.0060 (18)	0.0043 (18)
C321	0.0189 (19)	0.0164 (19)	0.018 (2)	−0.0054 (16)	−0.0061 (17)	−0.0040 (16)
C322	0.023 (2)	0.020 (2)	0.028 (2)	−0.0150 (17)	−0.0100 (18)	0.0005 (18)
C323	0.031 (2)	0.025 (2)	0.034 (3)	−0.0130 (19)	−0.021 (2)	−0.0015 (19)
C324	0.039 (3)	0.028 (2)	0.024 (2)	−0.015 (2)	−0.017 (2)	−0.0010 (19)
C325	0.029 (2)	0.028 (2)	0.020 (2)	−0.0143 (19)	−0.0038 (18)	−0.0028 (18)
C326	0.019 (2)	0.022 (2)	0.025 (2)	−0.0116 (17)	−0.0097 (18)	0.0013 (18)
C331	0.0132 (18)	0.0141 (19)	0.029 (2)	−0.0088 (16)	−0.0053 (17)	0.0019 (17)
C332	0.0147 (19)	0.019 (2)	0.024 (2)	−0.0083 (16)	−0.0003 (17)	−0.0004 (17)
C333	0.021 (2)	0.022 (2)	0.032 (3)	−0.0100 (18)	−0.0064 (19)	0.0106 (19)
C334	0.021 (2)	0.014 (2)	0.050 (3)	−0.0083 (18)	−0.003 (2)	0.005 (2)
C335	0.022 (2)	0.018 (2)	0.058 (3)	−0.0112 (18)	−0.007 (2)	−0.007 (2)
C336	0.018 (2)	0.020 (2)	0.036 (3)	−0.0098 (17)	−0.0068 (18)	−0.0051 (18)
C31	0.043 (3)	0.070 (4)	0.033 (3)	−0.038 (3)	−0.009 (2)	−0.003 (3)
C32	0.046 (3)	0.053 (3)	0.043 (3)	−0.018 (3)	−0.022 (3)	−0.005 (3)
C33	0.074 (4)	0.056 (3)	0.034 (3)	−0.042 (3)	−0.031 (3)	0.009 (3)
C34	0.054 (3)	0.092 (4)	0.035 (3)	−0.052 (3)	−0.012 (3)	−0.005 (3)
C35	0.034 (3)	0.071 (4)	0.072 (4)	−0.011 (3)	−0.024 (3)	−0.025 (3)
C36	0.080 (4)	0.052 (3)	0.067 (4)	−0.040 (3)	−0.050 (4)	0.015 (3)
C41	0.057 (4)	0.176 (8)	0.055 (4)	−0.076 (5)	−0.038 (4)	0.052 (5)
C42	0.091 (5)	0.098 (5)	0.067 (4)	−0.070 (4)	−0.054 (4)	0.050 (4)
C43	0.041 (3)	0.065 (4)	0.050 (3)	−0.024 (3)	−0.027 (3)	0.022 (3)
C44	0.045 (3)	0.070 (4)	0.036 (3)	−0.031 (3)	−0.025 (2)	0.013 (3)
C45	0.063 (4)	0.078 (4)	0.041 (3)	−0.013 (3)	−0.033 (3)	−0.003 (3)
C46	0.034 (3)	0.158 (7)	0.032 (3)	−0.018 (4)	−0.017 (3)	0.009 (4)
C61	0.091 (6)	0.127 (7)	0.166 (10)	−0.070 (6)	−0.082 (7)	0.036 (7)
C62	0.040 (3)	0.115 (6)	0.072 (5)	−0.023 (4)	−0.026 (3)	0.006 (5)
C63	0.037 (3)	0.110 (6)	0.065 (5)	−0.018 (4)	−0.027 (3)	0.023 (4)
C64	0.078 (5)	0.149 (8)	0.086 (6)	−0.039 (5)	−0.035 (5)	−0.014 (6)
C65	0.141 (9)	0.262 (15)	0.052 (6)	−0.127 (10)	−0.025 (5)	0.017 (8)
C66	0.156 (10)	0.270 (16)	0.114 (9)	−0.166 (12)	−0.069 (8)	0.084 (10)
C51A	0.059 (7)	0.096 (10)	0.056 (8)	−0.035 (7)	−0.026 (6)	−0.019 (7)
C52A	0.052 (6)	0.049 (6)	0.056 (7)	−0.016 (5)	−0.021 (5)	−0.012 (5)
C53A	0.026 (5)	0.060 (6)	0.027 (6)	−0.013 (4)	−0.005 (4)	−0.010 (5)
C54A	0.025 (7)	0.084 (9)	0.022 (7)	−0.022 (6)	−0.010 (6)	0.005 (7)
C55A	0.041 (6)	0.098 (10)	0.051 (8)	−0.033 (7)	−0.023 (5)	0.029 (7)
C56A	0.044 (8)	0.111 (13)	0.039 (8)	−0.031 (8)	−0.029 (7)	0.009 (8)
C51B	0.044 (7)	0.072 (9)	0.027 (7)	−0.025 (6)	−0.003 (5)	0.011 (6)
C52B	0.030 (9)	0.099 (13)	0.017 (8)	−0.018 (9)	0.000 (6)	−0.016 (9)
C53B	0.055 (7)	0.083 (10)	0.029 (7)	−0.035 (7)	−0.019 (6)	−0.001 (6)
C54B	0.081 (9)	0.064 (8)	0.052 (8)	−0.022 (7)	−0.043 (7)	0.000 (7)
C55B	0.051 (8)	0.119 (14)	0.046 (10)	−0.041 (9)	−0.026 (8)	0.006 (9)
C56B	0.062 (9)	0.092 (11)	0.059 (10)	−0.040 (8)	−0.039 (8)	0.008 (8)
C71	0.075 (15)	0.097 (18)	0.057 (14)	0.011 (15)	−0.029 (11)	0.013 (14)
C72	0.15 (3)	0.16 (3)	0.043 (14)	−0.09 (2)	−0.022 (17)	0.010 (15)
C73	0.22 (3)	0.18 (3)	0.039 (16)	−0.10 (3)	−0.002 (18)	0.014 (19)
C74	0.112 (19)	0.16 (3)	0.045 (12)	0.031 (18)	−0.013 (12)	0.031 (17)
C75	0.26 (5)	0.17 (3)	0.063 (18)	−0.12 (3)	−0.01 (2)	0.015 (19)
C76	0.060 (14)	0.26 (4)	0.052 (16)	−0.07 (2)	−0.011 (13)	0.01 (2)

### Geometric parameters (Å, °)

**Table:**

Pd1—P1	2.2825 (9)	C234—H234	0.9500
Pd1—Te4	2.5938 (4)	C235—C236	1.364 (5)
Pd1—Te3	2.6101 (3)	C235—H235	0.9500
Pd1—Te1	2.6268 (4)	C236—H236	0.9500
Pd2—P2	2.2937 (9)	C311—C312	1.394 (5)
Pd2—Te4	2.5928 (3)	C311—C316	1.396 (5)
Pd2—Te3	2.6101 (3)	C312—C313	1.383 (5)
Pd2—Te2	2.6389 (4)	C312—H312	0.9500
Pd3—P3	2.3001 (9)	C313—C314	1.370 (6)
Pd3—Te4	2.5928 (3)	C313—H313	0.9500
Pd3—Te1^i^	2.6124 (3)	C314—C315	1.382 (6)
Pd3—Te3	2.6125 (3)	C314—H314	0.9500
Te1—C11	2.139 (4)	C315—C316	1.381 (6)
Te1—Pd3^i^	2.6124 (3)	C315—H315	0.9500
Te2—C21	2.151 (4)	C316—H316	0.9500
Te3—Te4	3.2301 (3)	C321—C326	1.393 (5)
P1—C131	1.816 (4)	C321—C322	1.395 (5)
P1—C121	1.835 (3)	C322—C323	1.388 (5)
P1—C111	1.834 (4)	C322—H322	0.9500
P2—C231	1.823 (4)	C323—C324	1.382 (6)
P2—C221	1.826 (4)	C323—H323	0.9500
P2—C211	1.839 (4)	C324—C325	1.382 (5)
P3—C321	1.829 (4)	C324—H324	0.9500
P3—C331	1.836 (4)	C325—C326	1.382 (5)
P3—C311	1.837 (3)	C325—H325	0.9500
C11—C16	1.378 (6)	C326—H326	0.9500
C11—C12	1.389 (5)	C331—C332	1.378 (5)
C12—C13	1.387 (6)	C331—C336	1.401 (5)
C12—H12	0.9500	C332—C333	1.390 (5)
C13—C14	1.373 (7)	C332—H332	0.9500
C13—H13	0.9500	C333—C334	1.374 (6)
C14—C15	1.387 (6)	C333—H333	0.9500
C14—H14	0.9500	C334—C335	1.377 (7)
C15—C16	1.384 (5)	C334—H334	0.9500
C15—H15	0.9500	C335—C336	1.395 (6)
C16—H16	0.9500	C335—H335	0.9500
C21—C26	1.360 (6)	C336—H336	0.9500
C21—C22	1.383 (6)	C31—C32	1.3900
C22—C23	1.376 (6)	C31—C36	1.3900
C22—H22	0.9500	C31—H31	0.9500
C23—C24	1.375 (7)	C32—C33	1.3900
C23—H23	0.9500	C32—H32	0.9500
C24—C25	1.368 (7)	C33—C34	1.3900
C24—H24	0.9500	C33—H33	0.9500
C25—C26	1.449 (6)	C34—C35	1.3900
C25—H25	0.9500	C34—H34	0.9500
C26—H26	0.9500	C35—C36	1.3900
C111—C116	1.389 (5)	C35—H35	0.9500
C111—C112	1.400 (5)	C36—H36	0.9500
C112—C113	1.381 (6)	C41—C42	1.3900
C112—H112	0.9500	C41—C46	1.3900
C113—C114	1.379 (6)	C41—H41	0.9500
C113—H113	0.9500	C42—C43	1.3900
C114—C115	1.376 (6)	C42—H42	0.9500
C114—H114	0.9500	C43—C44	1.3900
C115—C116	1.384 (6)	C43—H43	0.9500
C115—H115	0.9500	C44—C45	1.3900
C116—H116	0.9500	C44—H44	0.9500
C121—C122	1.386 (5)	C45—C46	1.3900
C121—C126	1.400 (5)	C45—H45	0.9500
C122—C123	1.386 (5)	C46—H46	0.9500
C122—H122	0.9500	C61—C62	1.3900
C123—C124	1.370 (5)	C61—C66	1.3900
C123—H123	0.9500	C61—H61	0.9500
C124—C125	1.380 (5)	C62—C63	1.3900
C124—H124	0.9500	C62—H62	0.9500
C125—C126	1.385 (5)	C63—C64	1.3900
C125—H125	0.9500	C63—H63	0.9500
C126—H126	0.9500	C64—C65	1.3900
C131—C136	1.390 (5)	C64—H64	0.9500
C131—C132	1.391 (5)	C65—C66	1.3900
C132—C133	1.395 (5)	C65—H65	0.9500
C132—H132	0.9500	C66—H66	0.9500
C133—C134	1.375 (6)	C51A—C52A	1.3900
C133—H133	0.9500	C51A—C56A	1.3900
C134—C135	1.369 (6)	C51A—H51A	0.9500
C134—H134	0.9500	C52A—C53A	1.3900
C135—C136	1.390 (5)	C52A—H52A	0.9500
C135—H135	0.9500	C53A—C54A	1.3900
C136—H136	0.9500	C53A—H53A	0.9500
C211—C216	1.391 (5)	C54A—C55A	1.3900
C211—C212	1.405 (5)	C54A—H54A	0.9500
C212—C213	1.384 (5)	C55A—C56A	1.3900
C212—H212	0.9500	C55A—H55A	0.9500
C213—C214	1.379 (6)	C56A—H56A	0.9500
C213—H213	0.9500	C51B—C52B	1.3900
C214—C215	1.361 (7)	C51B—C56B	1.3900
C214—H214	0.9500	C51B—H51B	0.9500
C215—C216	1.395 (6)	C52B—C53B	1.3900
C215—H215	0.9500	C52B—H52B	0.9500
C216—H216	0.9500	C53B—C54B	1.3900
C221—C222	1.398 (5)	C53B—H53B	0.9500
C221—C226	1.404 (5)	C54B—C55B	1.3900
C222—C223	1.390 (6)	C54B—H54B	0.9500
C222—H222	0.9500	C55B—C56B	1.3900
C223—C224	1.369 (6)	C55B—H55B	0.9500
C223—H223	0.9500	C56B—H56B	0.9500
C224—C225	1.383 (6)	C71—C72	1.3900
C224—H224	0.9500	C71—C76	1.3900
C225—C226	1.393 (6)	C71—H71	0.9500
C225—H225	0.9500	C72—C73	1.3900
C226—H226	0.9500	C72—H72	0.9500
C231—C232	1.382 (5)	C73—C74	1.3900
C231—C236	1.401 (5)	C73—H73	0.9500
C232—C233	1.390 (5)	C74—C75	1.3900
C232—H232	0.9500	C74—H74	0.9500
C233—C234	1.379 (6)	C75—C76	1.3900
C233—H233	0.9500	C75—H75	0.9500
C234—C235	1.374 (6)	C76—H76	0.9500
			
P1—Pd1—Te4	95.55 (3)	C232—C231—P2	117.4 (3)
P1—Pd1—Te3	172.28 (3)	C236—C231—P2	123.4 (3)
Te4—Pd1—Te3	76.735 (10)	C231—C232—C233	120.6 (4)
P1—Pd1—Te1	91.20 (3)	C231—C232—H232	119.7
Te4—Pd1—Te1	170.035 (13)	C233—C232—H232	119.7
Te3—Pd1—Te1	96.480 (10)	C234—C233—C232	119.5 (4)
P2—Pd2—Te4	96.57 (3)	C234—C233—H233	120.3
P2—Pd2—Te3	169.46 (3)	C232—C233—H233	120.3
Te4—Pd2—Te3	76.752 (10)	C235—C234—C233	120.1 (4)
P2—Pd2—Te2	101.95 (3)	C235—C234—H234	119.9
Te4—Pd2—Te2	161.133 (13)	C233—C234—H234	119.9
Te3—Pd2—Te2	85.393 (11)	C236—C235—C234	120.8 (4)
P3—Pd3—Te4	93.92 (2)	C236—C235—H235	119.6
P3—Pd3—Te1^i^	101.34 (2)	C234—C235—H235	119.6
Te4—Pd3—Te1^i^	164.420 (13)	C235—C236—C231	120.2 (4)
P3—Pd3—Te3	170.63 (3)	C235—C236—H236	119.9
Te4—Pd3—Te3	76.711 (10)	C231—C236—H236	119.9
Te1^i^—Pd3—Te3	88.017 (10)	C312—C311—C316	118.4 (3)
C11—Te1—Pd3^i^	109.22 (9)	C312—C311—P3	120.2 (3)
C11—Te1—Pd1	98.82 (10)	C316—C311—P3	120.9 (3)
Pd3^i^—Te1—Pd1	108.806 (11)	C313—C312—C311	120.7 (4)
C21—Te2—Pd2	113.88 (10)	C313—C312—H312	119.7
Pd2—Te3—Pd1	82.639 (10)	C311—C312—H312	119.7
Pd2—Te3—Pd3	92.981 (11)	C314—C313—C312	120.0 (4)
Pd1—Te3—Pd3	78.618 (10)	C314—C313—H313	120.0
Pd2—Te3—Te4	51.384 (8)	C312—C313—H313	120.0
Pd1—Te3—Te4	51.407 (8)	C313—C314—C315	120.4 (4)
Pd3—Te3—Te4	51.371 (8)	C313—C314—H314	119.8
Pd3—Te4—Pd2	93.845 (11)	C315—C314—H314	119.8
Pd3—Te4—Pd1	79.270 (10)	C316—C315—C314	120.0 (4)
Pd2—Te4—Pd1	83.292 (11)	C316—C315—H315	120.0
Pd3—Te4—Te3	51.918 (8)	C314—C315—H315	120.0
Pd2—Te4—Te3	51.865 (8)	C315—C316—C311	120.5 (4)
Pd1—Te4—Te3	51.859 (8)	C315—C316—H316	119.7
C131—P1—C121	102.66 (15)	C311—C316—H316	119.7
C131—P1—C111	105.90 (17)	C326—C321—C322	119.2 (4)
C121—P1—C111	102.90 (16)	C326—C321—P3	118.3 (3)
C131—P1—Pd1	110.95 (12)	C322—C321—P3	122.5 (3)
C121—P1—Pd1	117.64 (12)	C323—C322—C321	119.9 (4)
C111—P1—Pd1	115.40 (11)	C323—C322—H322	120.0
C231—P2—C221	107.31 (17)	C321—C322—H322	120.0
C231—P2—C211	99.87 (17)	C324—C323—C322	120.4 (4)
C221—P2—C211	100.78 (17)	C324—C323—H323	119.8
C231—P2—Pd2	111.61 (12)	C322—C323—H323	119.8
C221—P2—Pd2	116.58 (12)	C325—C324—C323	119.8 (4)
C211—P2—Pd2	118.77 (12)	C325—C324—H324	120.1
C321—P3—C331	106.17 (17)	C323—C324—H324	120.1
C321—P3—C311	103.31 (16)	C326—C325—C324	120.4 (4)
C331—P3—C311	103.02 (16)	C326—C325—H325	119.8
C321—P3—Pd3	114.63 (12)	C324—C325—H325	119.8
C331—P3—Pd3	114.58 (12)	C325—C326—C321	120.3 (4)
C311—P3—Pd3	113.83 (11)	C325—C326—H326	119.8
C16—C11—C12	119.6 (4)	C321—C326—H326	119.8
C16—C11—Te1	123.2 (3)	C332—C331—C336	118.9 (4)
C12—C11—Te1	117.2 (3)	C332—C331—P3	117.7 (3)
C13—C12—C11	119.9 (4)	C336—C331—P3	123.4 (3)
C13—C12—H12	120.1	C331—C332—C333	121.6 (4)
C11—C12—H12	120.1	C331—C332—H332	119.2
C14—C13—C12	120.5 (4)	C333—C332—H332	119.2
C14—C13—H13	119.8	C334—C333—C332	119.1 (4)
C12—C13—H13	119.8	C334—C333—H333	120.5
C13—C14—C15	119.5 (4)	C332—C333—H333	120.5
C13—C14—H14	120.2	C333—C334—C335	120.6 (4)
C15—C14—H14	120.2	C333—C334—H334	119.7
C16—C15—C14	120.3 (4)	C335—C334—H334	119.7
C16—C15—H15	119.9	C334—C335—C336	120.5 (4)
C14—C15—H15	119.9	C334—C335—H335	119.7
C11—C16—C15	120.1 (4)	C336—C335—H335	119.7
C11—C16—H16	119.9	C335—C336—C331	119.3 (4)
C15—C16—H16	119.9	C335—C336—H336	120.4
C26—C21—C22	120.5 (4)	C331—C336—H336	120.4
C26—C21—Te2	119.1 (3)	C32—C31—C36	120.0
C22—C21—Te2	120.3 (3)	C32—C31—H31	120.0
C23—C22—C21	120.8 (5)	C36—C31—H31	120.0
C23—C22—H22	119.6	C31—C32—C33	120.0
C21—C22—H22	119.6	C31—C32—H32	120.0
C24—C23—C22	121.0 (5)	C33—C32—H32	120.0
C24—C23—H23	119.5	C34—C33—C32	120.0
C22—C23—H23	119.5	C34—C33—H33	120.0
C25—C24—C23	118.6 (4)	C32—C33—H33	120.0
C25—C24—H24	120.7	C35—C34—C33	120.0
C23—C24—H24	120.7	C35—C34—H34	120.0
C24—C25—C26	121.3 (4)	C33—C34—H34	120.0
C24—C25—H25	119.4	C34—C35—C36	120.0
C26—C25—H25	119.4	C34—C35—H35	120.0
C21—C26—C25	117.7 (4)	C36—C35—H35	120.0
C21—C26—H26	121.1	C35—C36—C31	120.0
C25—C26—H26	121.1	C35—C36—H36	120.0
C116—C111—C112	118.2 (4)	C31—C36—H36	120.0
C116—C111—P1	124.9 (3)	C42—C41—C46	120.0
C112—C111—P1	116.9 (3)	C42—C41—H41	120.0
C113—C112—C111	120.7 (4)	C46—C41—H41	120.0
C113—C112—H112	119.6	C43—C42—C41	120.0
C111—C112—H112	119.6	C43—C42—H42	120.0
C114—C113—C112	120.2 (4)	C41—C42—H42	120.0
C114—C113—H113	119.9	C42—C43—C44	120.0
C112—C113—H113	119.9	C42—C43—H43	120.0
C115—C114—C113	119.6 (4)	C44—C43—H43	120.0
C115—C114—H114	120.2	C45—C44—C43	120.0
C113—C114—H114	120.2	C45—C44—H44	120.0
C114—C115—C116	120.6 (4)	C43—C44—H44	120.0
C114—C115—H115	119.7	C46—C45—C44	120.0
C116—C115—H115	119.7	C46—C45—H45	120.0
C115—C116—C111	120.6 (4)	C44—C45—H45	120.0
C115—C116—H116	119.7	C45—C46—C41	120.0
C111—C116—H116	119.7	C45—C46—H46	120.0
C122—C121—C126	118.5 (3)	C41—C46—H46	120.0
C122—C121—P1	122.6 (3)	C62—C61—C66	120.0
C126—C121—P1	118.8 (3)	C62—C61—H61	120.0
C121—C122—C123	120.6 (4)	C66—C61—H61	120.0
C121—C122—H122	119.7	C61—C62—C63	120.0
C123—C122—H122	119.7	C61—C62—H62	120.0
C124—C123—C122	120.5 (4)	C63—C62—H62	120.0
C124—C123—H123	119.7	C64—C63—C62	120.0
C122—C123—H123	119.7	C64—C63—H63	120.0
C123—C124—C125	119.8 (3)	C62—C63—H63	120.0
C123—C124—H124	120.1	C65—C64—C63	120.0
C125—C124—H124	120.1	C65—C64—H64	120.0
C124—C125—C126	120.3 (3)	C63—C64—H64	120.0
C124—C125—H125	119.8	C64—C65—C66	120.0
C126—C125—H125	119.8	C64—C65—H65	120.0
C125—C126—C121	120.3 (3)	C66—C65—H65	120.0
C125—C126—H126	119.9	C65—C66—C61	120.0
C121—C126—H126	119.9	C65—C66—H66	120.0
C136—C131—C132	118.3 (3)	C61—C66—H66	120.0
C136—C131—P1	120.3 (3)	C52A—C51A—C56A	120.0
C132—C131—P1	121.3 (3)	C52A—C51A—H51A	120.0
C131—C132—C133	120.3 (4)	C56A—C51A—H51A	120.0
C131—C132—H132	119.9	C51A—C52A—C53A	120.0
C133—C132—H132	119.9	C51A—C52A—H52A	120.0
C134—C133—C132	120.1 (4)	C53A—C52A—H52A	120.0
C134—C133—H133	120.0	C52A—C53A—C54A	120.0
C132—C133—H133	120.0	C52A—C53A—H53A	120.0
C135—C134—C133	120.4 (4)	C54A—C53A—H53A	120.0
C135—C134—H134	119.8	C55A—C54A—C53A	120.0
C133—C134—H134	119.8	C55A—C54A—H54A	120.0
C134—C135—C136	119.8 (4)	C53A—C54A—H54A	120.0
C134—C135—H135	120.1	C56A—C55A—C54A	120.0
C136—C135—H135	120.1	C56A—C55A—H55A	120.0
C131—C136—C135	121.1 (3)	C54A—C55A—H55A	120.0
C131—C136—H136	119.5	C55A—C56A—C51A	120.0
C135—C136—H136	119.5	C55A—C56A—H56A	120.0
C216—C211—C212	118.2 (4)	C51A—C56A—H56A	120.0
C216—C211—P2	122.0 (3)	C52B—C51B—C56B	120.0
C212—C211—P2	119.7 (3)	C52B—C51B—H51B	120.0
C213—C212—C211	120.2 (4)	C56B—C51B—H51B	120.0
C213—C212—H212	119.9	C53B—C52B—C51B	120.0
C211—C212—H212	119.9	C53B—C52B—H52B	120.0
C214—C213—C212	120.8 (4)	C51B—C52B—H52B	120.0
C214—C213—H213	119.6	C52B—C53B—C54B	120.0
C212—C213—H213	119.6	C52B—C53B—H53B	120.0
C215—C214—C213	119.6 (4)	C54B—C53B—H53B	120.0
C215—C214—H214	120.2	C55B—C54B—C53B	120.0
C213—C214—H214	120.2	C55B—C54B—H54B	120.0
C214—C215—C216	120.9 (4)	C53B—C54B—H54B	120.0
C214—C215—H215	119.6	C54B—C55B—C56B	120.0
C216—C215—H215	119.6	C54B—C55B—H55B	120.0
C211—C216—C215	120.3 (4)	C56B—C55B—H55B	120.0
C211—C216—H216	119.8	C55B—C56B—C51B	120.0
C215—C216—H216	119.8	C55B—C56B—H56B	120.0
C222—C221—C226	118.7 (4)	C51B—C56B—H56B	120.0
C222—C221—P2	122.8 (3)	C72—C71—C76	120.0
C226—C221—P2	118.4 (3)	C72—C71—H71	120.0
C223—C222—C221	119.8 (4)	C76—C71—H71	120.0
C223—C222—H222	120.1	C73—C72—C71	120.0
C221—C222—H222	120.1	C73—C72—H72	120.0
C224—C223—C222	121.2 (4)	C71—C72—H72	120.0
C224—C223—H223	119.4	C74—C73—C72	120.0
C222—C223—H223	119.4	C74—C73—H73	120.0
C223—C224—C225	119.9 (4)	C72—C73—H73	120.0
C223—C224—H224	120.1	C75—C74—C73	120.0
C225—C224—H224	120.1	C75—C74—H74	120.0
C224—C225—C226	120.1 (4)	C73—C74—H74	120.0
C224—C225—H225	119.9	C74—C75—C76	120.0
C226—C225—H225	119.9	C74—C75—H75	120.0
C225—C226—C221	120.2 (4)	C76—C75—H75	120.0
C225—C226—H226	119.9	C75—C76—C71	120.0
C221—C226—H226	119.9	C75—C76—H76	120.0
C232—C231—C236	118.8 (3)	C71—C76—H76	120.0

Symmetry codes: (i) −*x*+1, −*y*, −*z*.
